# Encapsulation of EGCG by Zein-Gum Arabic Complex Nanoparticles and In Vitro Simulated Digestion of Complex Nanoparticles

**DOI:** 10.3390/foods11142131

**Published:** 2022-07-19

**Authors:** Jianchang Jin, Chengzhi Liu, Huafei Tong, Yulu Sun, Min Huang, Gerui Ren, Hujun Xie

**Affiliations:** 1 College of Biological and Environmental Engneering, Zhejiang Shuren University, Hangzhou 310015, China; jinjianchang2004@163.com; 2 School of Food Science and Biotechnology, Zhejiang Gongshang University, Hangzhou 310018, China; liucz0815@163.com (C.L.); thf163yx@163.com (H.T.); s2461906402@163.com (Y.S.); huangmin@zjgsu.edu.cn (M.H.); rengerui@zjgsu.edu.cn (G.R.)

**Keywords:** zein, gum arabic, EGCG, encapsulation, complex nanoparticles

## Abstract

Epigallocatechin gallate (EGCG) has many excellent qualities such as its antitumor, antiradiation and anti-oxidation properties, but its application is limited because its oral bioavailability is low and stability is poor. In this paper, zein and gum arabic (GA) were used as wall materials to prepare Zein-GA complex nanoparticles for encapsulating and protecting the EGCG. The particle size of Zein-GA-EGCG complex nanoparticles ranged from 128.03–221.23 nm, and the EGCG encapsulation efficiency reached a maximum of 75.23% when the mass ratio of zein to GA was 1:1. The FTIR and XRD results illustrated that the components of the Zein-GA-EGCG complex nanoparticles interacted by electrostatic, hydrogen bonding, and hydrophobic interactions. The EGCG release rate of Zein-GA-EGCG nanoparticles (16.42%) was lower than that of Zein-EGCG (25.52%) during gastric digestion, and a large amount of EGCG was released during intestinal digestion, suggesting that the Zein-GA-EGCG nanoparticles could achieve the sustained release of EGCG during in vitro digestion. Hence, using Zein-GA complexes to encapsulate EGCG effectively increased the encapsulation efficiency of EGCG and realized the purpose of sustained release during simulated gastrointestinal digestion.

## 1. Introduction

Epigallocatechin gallate (EGCG) is a kind of polyphenol from tea. Recent studies have shown that EGCG has many excellent properties such as antitumor, antiradiation, and anti-oxidation capability [1,2,3,4,5,6]. However, EGCG is sensitive to neutral and alkaline solutions and becomes unstable as the oxygen concentration, temperature, and pH increases, resulting in impossible direct usage in these environments [7,8,9,10]. In addition, EGCG is unstable in the gastrointestinal environment, leading to low bioavailability [11]. Therefore, the development of functional foods based on EGCG is faced with many challenges. Multiple delivery systems have been developed to improve the stability and bioavailability of EGCG. Gulseren et al. constructed nanoliposomes using milk phospholipids and soybean phospholipids, respectively, to protect EGCG, and the results revealed that the behaviors of these two vesicles were different although both of them showed high bioefficacy and delivery of polyphenols [12]. Liang et al. constructed chitosan nanocapsules using modified carboxymethyl chitosan and chitosan hydrochloride as raw materials to encapsulate EGCG, and the results proved that these materials improved the sustained release of EGCG [13]. Chen et al. encapsulated EGCG in an emulsion gel composed of sucrose, gelatin, polyglycerol fatty acid ester, and gliadin, and the results showed that the emulsion gel improved the chemical stability of EGCG [14].

In recent years, protein–polysaccharide complexes as transport carriers for nutrients have become a research hotspot because these complexes often show better functional characteristics than protein or polysaccharide alone. However, how to select the appropriate proteins and polysaccharides to form the delivery system remains to be explored. Zein is a storage protein found in corn [15]. It is insoluble in water, but soluble in ethanol aqueous solution, high-concentration urea, strong acid or strong alkali, and other solvents. Zein has this unique solubility because its constituent components contain more than 50% hydrophobic amino acids [16]. Zein has good biocompatibility, biodegradability, and unique self-assembly, which make it be widely used as a transport carrier for bioactive substances. Liang et al. constructed a composite system of Zein-HTCC to encapsulate curcumin, and the results suggested that the chemical stability of curcumin was enhanced significantly [17]. Zhang et al. prepared the zein-caseinate-alginate complex nanoparticles loaded with propolis by the method of one-step preparation. The results revealed that the encapsulation efficiency of propolis by nanoparticles reached to 86.5%, and the bioavailability of the encapsulated propolis increased to 80% compared with free propolis [18]. Gum arabic (GA) is a kind of amphiphilic polysaccharide with complex molecular structure, and it is widely used in the food industry because of its good emulsification, thickening ability, and low digestibility [19]. Thus, GA has the ability to improve the stability of a protein delivery system [20,21].

In this study, Zein-GA-EGCG complex nanoparticles were prepared using zein and acacia gum as wall materials. The objective of this work was to investigate the effects of mass ratio of zein to GA on particle size and encapsulation efficiency, analyze the release behavior of EGCG during in vitro digestion, and preliminarily explore the interaction mechanism between zein, GA and EGCG.

## 2. Materials and Methods

### 2.1. Materials

(−)-Epigallocatechin gallate (EGCG, HPLC ≥98%) were purchased from Shanghai Yuanye Bio-Technology Co., Ltd. (Shanghai, China). Gum arabic, pepsin (porcine, 1:3000), and pancreatin from porcine pancreas (USP) were purchased from Aladdin Co., Ltd. (Shanghai, China). Zein was obtained from Sigma-Aldrich Corp (St. Louis, MO, USA). Other chemicals were all analytical grade and purchased from Aladdin Co., Ltd. (Shanghai, China).

### 2.2. Preparation of Zein-GA-EGCG (E-Z/G) Samples

The Zein-EGCG complex nanoparticles were fabricated by the anti-solvent precipitation method. Zein (0.1 g) and EGCG (0.01 g) were dissolved in 20 mL ethanol-water solution (80%, *v*/*v*). After stirring for 60 min at 400 rpm, the 20 mL Zein-EGCG aqueous-ethanol solution was dropped into 50 mL deionized water to form dispersions. The dispersions were adjusted to pH 4.0 using 0.1 mol/L NaOH or HCl. Ethanol in the system was removed using rotary evaporation at 45 °C under vacuum, and the loss of ethanol was compensated using deionized water to form Zein-EGCG complex nanoparticles.

The GA of different masses (0.200, 0.150, 0.100, 0.050, 0.033, 0.020 g) was dissolved in 46 mL ultrapure water and stirred with magnetic agitator for 2 h. The pH of GA solutions was adjusted to 4.0 using 0.1 mol/L HCl or NaOH. Then, Zein-EGCG complex nanoparticles were slowly added into the GA solutions with continuous stirring at 400 rpm for 2 h to form Zein-GA-EGCG complex nanoparticles. The mass ratios of zein to GA were 1:2, 2:3, 1:1, 2:1, 3:1 and 5:1 (*w*/*w*), which were denoted as E-Z/G_1:2_, E-Z/G_2:3_, E-Z/G_1:1_, E-Z/G_2:1_,E-Z/G_3:1_ and E-Z/G_5:1_, respectively. The freshly prepared samples were stored at 4 °C for subsequent analysis, and the solid samples were obtained by freeze-drying.

### 2.3. The Particle Size, Polydispersity Index (PDI) and Zeta Potential of Complex Nanoparticles

The particle size and zeta potential of Zein-EGCG and Zein-GA-EGCG complex nanoparticles with different Zein/GA ratios were measured at room temperature by nano-ZS laser particle sizer (Malvern, UK). All samples were tested in parallel three times.

### 2.4. Encapsulation Efficiency (EE) and Loading Capacity (LC) Measurement

EGCG reserve solution was obtained through dissolving a certain amount of EGCG powder in ultrapure water. A series of EGCG solutions in different concentrations were obtained by dilution. The standard curve of EGCG was drawn by testing the absorbance at 274 nm.

Sample solutions were centrifuged for 20 min at 4000 rpm in an ultrafiltration tube with a molecular weight of 3 k, and the content of EGCG in the supernatant was measured as described above. The encapsulation efficiency (*EE*) and loading capacity (*LC*) were obtained from Equations (1) and (2), respectively.

(1) $$EE(\%)\;=\frac{\mathrm{Total}\;\mathrm{Amount}\;\mathrm{of}\;\mathrm{EGCG}-\mathrm{Amount}\;\mathrm{of}\;\mathrm{Free}\;\mathrm{EGCG}}{\mathrm{Total}\;\mathrm{Amount}\;\mathrm{of}\;\mathrm{EGCG}}\times 100$$

(2) $$LC(\%)=\frac{\mathrm{Total}\;\mathrm{Amount}\;\mathrm{of}\;\mathrm{EGCG}-\mathrm{Amount}\;\mathrm{of}\;\mathrm{Free}\;\mathrm{EGCG}}{\mathrm{Total}\;\mathrm{Amount}\;\mathrm{of}\;\mathrm{Sample}}\times 100$$

where total amount of EGCG is the total mass of EGCG contained in the complex nanoparticle suspension, the amount of free EGCG is the mass of EGCG obtained in the filtrate receiver after ultrafiltration using ultrafiltration tubes. The total amount of sample is the total mass of zein and GA.

### 2.5. Morphology Characterization of Complex Nanoparticles

A scanning electron microscopy (SU8010, Hitachi, Tokyo, Japan) was applied to obtain the micromorphology of zein, Zein-EGCG and Zein-GA-EGCG complex nanoparticles. The samples were treated with gold spraying before observation. The acceleration voltage was set as 3 kV.

### 2.6. Fourier Transform Infrared Spectroscopy (FTIR)

The samples were centrifuged by using the ultrafiltration tubes to remove the free EGCG in supernatant before freeze-drying. A Fourier infrared spectrometer (Nicolet iS5, Thermo Fisher, New York, NY, USA) was applied to analyze the zein, GA, EGCG, Zein-EGCG and Zein-GA-EGCG complex nanoparticles. All samples were scanned 64 times, the resolution was 4 cm^−1^, and the scanning range was 4000–400 cm^−1^.

### 2.7. X-ray Diffraction (XRD) Test

The samples were centrifuged by using the ultrafiltration tubes to remove the free EGCG in supernatant before freeze-drying. An X-ray diffractometer (Brucker D8 advance, Bruker, Karlsruhe, Germany) was applied to analyze the zein, GA, EGCG, Zein-EGCG and different Zein-GA-EGCG samples. The scanning was performed at a frequency of 8°/min in the range of 0–80° (2θ) at 20 °C with a working current of 100 mA and a voltage of 40 kV.

### 2.8. Differential Scanning Calorimetry (DSC)

A differential scanning calorimeter (EVO 131, Setaram Instrumentation, Savoie, France) was employed to analyze the thermal properties of zein, GA and EGCG-loaded complex nanoparticles. During the experiment, the nitrogen flow was 20 mL/min, the heating rate was 10 °C/min in the range of 30~200 °C.

### 2.9. Antioxidant Activity Test

The DPPH free radical scavenging ability of zein, GA and EGCG-loaded complex nanoparticles was evaluated according to Yi et al. [22]. Before the DPPH experiment, the freshly prepared samples were evenly divided into three parts, one was without any treatment, one was adjusted pH to 6.8, and one was heated in a water bath pot (60 °C) for 1 h. DPPH solution (0.1 mmol/L) was obtained using anhydrous ethanol. The sample was fully mixed with DPPH solution in equal volume and stored in dark for 30 min and labeled as *A*_1_ of sample group. The blank group was prepared by mixing the sample and ethanol-aqueous solution in equal volume and was labeled as *A*_2_ of blank group. The control group was prepared by mixing DPPH solution and distilled water in equal volume and labeled as *A*_0_. The absorbance of all samples was tested by an ultraviolet spectrophotometer (UV-2600, Shimadzu, Kyoto, Japan) at 537 nm. DPPH free radical scavenging capacity was calculated by the Formula (3).

(3) $$\mathrm{DPPH}\;\mathrm{Scavenging}\;\mathrm{Activity}\;(\%)=\left(1-\frac{A_{1}-A_{2}}{A_{0}}\right)\times 100$$

The ABTS free radical scavenging ability of zein, GA and EGCG-loaded complex nanoparticles were tested according to Liu et al. [23]. Before the ABTS experiment, the freshly prepared samples were evenly divided into three parts, one was without any treatment, one was adjusted pH to 6.8, and one was heated in a water bath pot (60 °C) for 1 h. ABTS radical solution was obtained by mixing ABTS solution (7 mM) with potassium persulfate solution (4.9 mM) for 12 h in a dark environment. The sample of 40 μL was mixed with ABTS solution of 4 mL, which had been diluted to the absorbance of 0.70 at 734 nm before using. The Equation (4) was used to calculate the data.

(4) $$\mathrm{ABTS}\;\mathrm{Scavenging}\;\mathrm{Activity}\;(\%)=\frac{A_{C}-A_{S}}{A_{C}}\times 100$$

where *A_C_* is the absorbance value of ABTS mixed with anhydrous ethanol, *A_S_* is the absorbance value of ABTS mixed with the sample.

### 2.10. In Vitro Release Study

The in vitro mimic digestion was conducted based on the method of Liang et al. [24]. The simulated gastric fluid (SGF) was prepared as follows. Pepsin (0.800 g) and NaCl (0.500 g) were dissolved in some deionized water. After stirring for 60 min at 400 rpm, the solution was added to with 1.75 mL 12 M HCl. The deionized water was used to fix the volume to 250 mL. The simulated intestinal fluid (SIF) was prepared as follows. Bile salt (1.250 g, the mass ratio of sodium cholate to sodium deoxycholate was 1:1), NaCl (0.450 g), pancreatin (2.000 g) and CaCl_2_ (0.008 g) were dissolved in deionized water. After fully dissolving, the deionized water was used to fix the volume to 250 mL.

Zein-EGCG and Zein-GA-EGCG sample solutions were mixed with the SGF and SIF, respectively. The ratio of sample to gastroenteric fluid was 1:1. The pH was adjusted to 2.0 for gastric digestion and 7.0 for intestional digestion. The simulated gastric digestion and the simulated intestinal digestion were performed in a shaker at 37 °C with 100 rpm and at 37 °C with 270 rpm, respectively. The EGCG cumulative release rate of Zein-GA-EGCG was obtained by the following formula:

(5) $$\mathrm{Cumulative}\;\mathrm{Release}\;\mathrm{Rate}\;\left(\%\right)=\frac{C\times V}{M}\times 100$$

where *C* represents the EGCG concentration released from the mixed solution (mg/L), *V* represents the volume (mL) of the solution, and *M* represents the amount of EGCG included in complex nanoparticles (mg).

### 2.11. Statistical Analysis

Each sample was measured three times, and the results are shown as mean ± standard deviation. Statistical differences were analyzed by ANOVA with Duncan’s post hoc test. The significance level was *p* < 0.05.

## 3. Results and Discussion

### 3.1. Analysis of Particle Size, Zeta Potential and Encapsulation Efficiency

**Figure 1:**
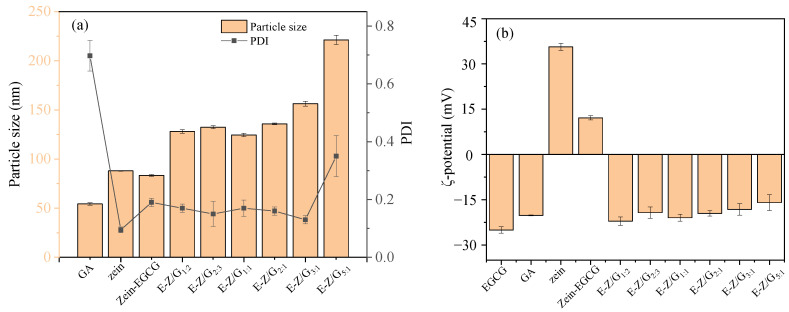
The particle size and PDI of different samples (**a**), and the zeta potential of different samples (**b**).

The effect of GA content on the zeta potential, particle size, and polydispersion index (PDI) of Zein-GA-EGCG is shown in Figure 1. The particle size of zein particles encapsulated with EGCG was 82.77 nm, which was in line with the previous results of zein nanoparticles loaded with pterostilbene [25]. The particle size of Zein-GA-EGCG complex nanoparticle (E-Z/G) samples decreased significantly as values of zein:GA changed from 5:1 to 1:1, which might be due to the interaction between Zein-EGCG and GA. By increasing the GA content, more electrostatic interactions were formed and the Zein-GA-EGCG complex nanoparticles were made to bind more closely, resulting in the smaller particle size [26]. The particle size of complex nanoparticles increased slightly as values of zein:GA changed to 1:2, which might be due to excessive GA producing crosslinking between complex nanoparticles, resulting in an increase in particle size [25]. A similar result was also obtained in the zein-hyaluronic acid nanoparticle system [27].

The zeta potential of Zein-EGCG was positive (12.13 mV, Figure 1). This was because there was far more positively charged zein (35.65 mV) than negatively charged EGCG (−25.05 mV) in the Zein-EGCG nanoparticle system at pH 4, leading to the overall positive charge of the nanoparticles. With the increase in GA content, the negative charge on the surface of nanoparticles increased and the electrostatic repulsion between the nanoparticles increased Therefore, the Zein-GA-EGCG complex nanoparticles formed a relatively dense structure, resulting in a reduction in particle size. The experimental results showed that GA was adsorbed on the surface of zein nanoparticles by electrostatic attraction, and the negatively charged GA stabilized the nanoparticles by electrostatic and steric repulsion [25,28]. In addition, the PDI values of E-Z/G complex nanoparticles were less than 0.4, indicating that E-Z/G complex nanoparticles were evenly dispersed in the solution.

**Figure 2:**
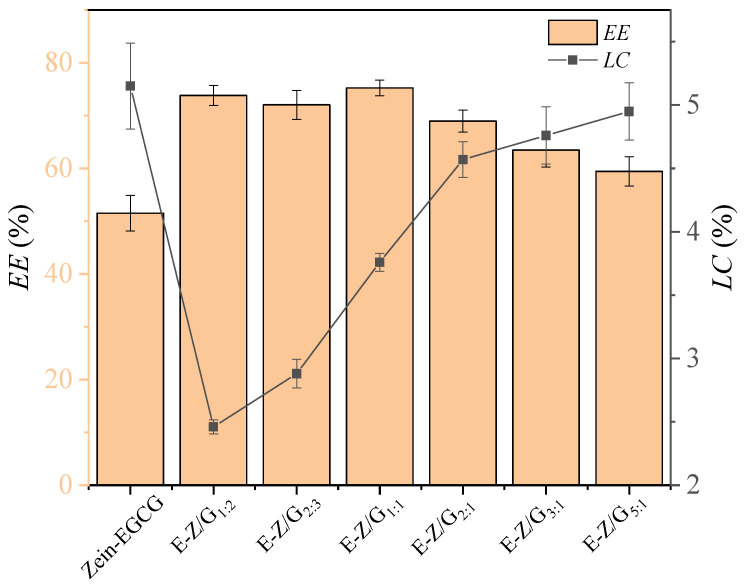
The *EE* and *LC* values of different samples.

Encapsulation efficiency of food functional compounds is one of the main factors determining the functional characteristics of a food delivery system. The encapsulation efficiency of zein for EGCG was 51.52% (Figure 2), which was in line with the results of Donsi et al. [29]. The *EE* values of EGCG in Zein-GA-EGCG nanoparticles increased significantly with increasing GA. The *EE* values reached the highest (75.23%) at the mass ratio of zein:GA = 1:1. The appearance of GA significantly increased the encapsulation efficiency of EGCG, which might be because GA can promote the entry of free EGCG or EGCG attached to zein into the complex nanoparticles. In addition, the *LC* of EGCG decreased gradually when values of zein:GA changed from 5:1 to 1:2, this was mainly because with the mass increase in GA, the mass change of EGCG encapsulated in complex nanoparticles was lower than that of the carrier.

### 3.2. Microscopic Morphology Characterization

**Figure 3:**
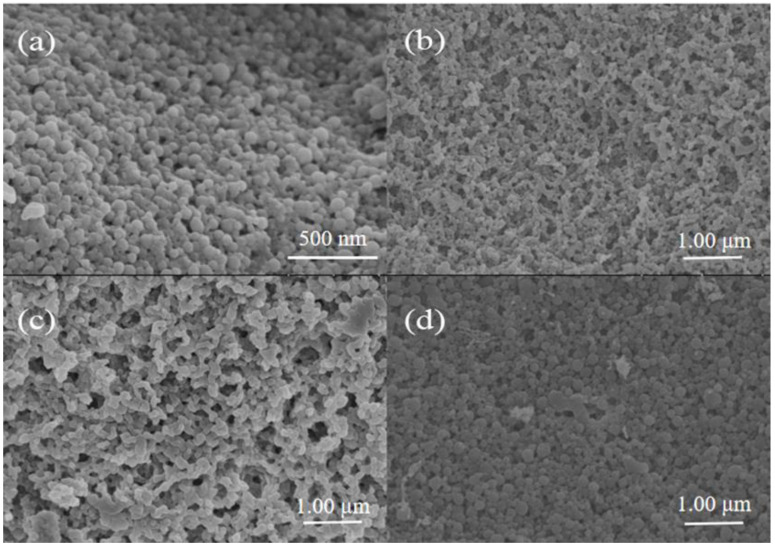
Microscopic morphology of different complex nanoparticles. (**a**) Zein-EGCG complex nanoparticles; (**b**) E-Z/G_1:1_ complex nanoparticles; (**c**) E-Z/G_5:1_ complex nanoparticles; (**d**) E-Z/G_2:3_ complex nanoparticles.

Figure 3 shows the microstructure images of Zein-EGCG, E-Z/G_1:1_, E-Z/G_5:1_ and E-Z/G_2:3_ samples. The Zein-EGCG sample was uniformly spherical with small particle size (Figure 3a). The addition of GA influenced the shape of the complex nanoparticles. When the GA content was low (Figure 3c), adhesion occurred between the complex nanoparticles. The relatively low surface charge of E-Z/G_5:1_ might have led to insufficient electrostatic repulsion, which made the complex nanoparticles adhere to each other. However, the size of the nanoparticles became uneven and adhesion also occurred in the sample of E-Z/G_2:3_ (Figure 3d), which might have been caused by excess GA binding on the surface of complex nanoparticles [25]. The size of the complex nanoparticles became relatively uniform in the E-Z/G_1:1_ sample (Figure 3b). In general, these results were in line with the results of particle size.

### 3.3. Analysis of FTIR

**Figure 4:**
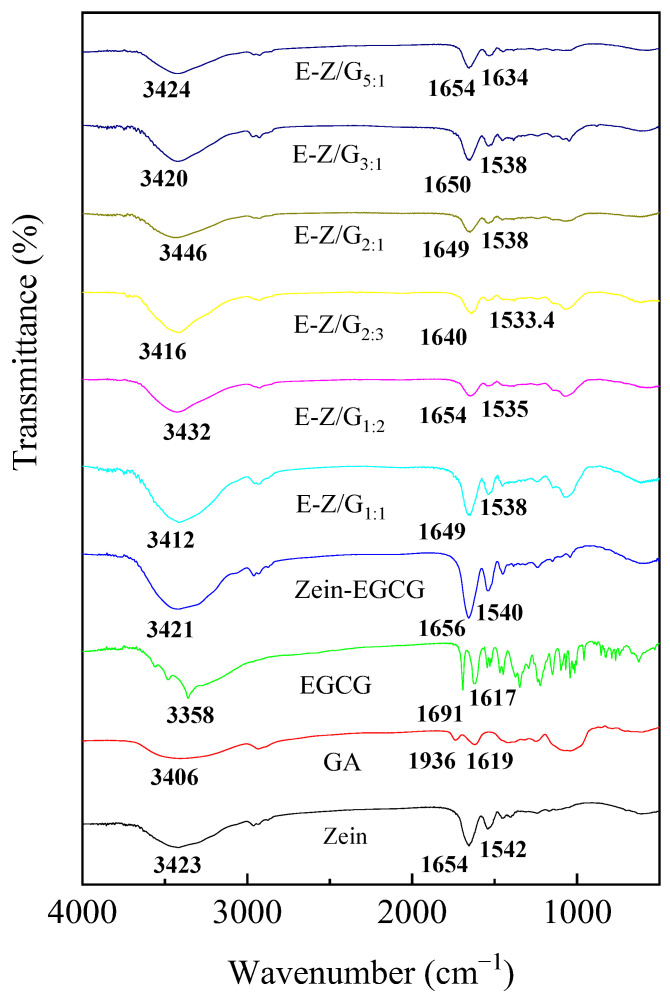
The infrared spectra of different samples.

FTIR was used to analyze the interaction between the different components (Figure 4). The band of 3200–3500 cm^−1^ was the stretching vibration of O–H bond, and the characteristic absorption peaks of zein, EGCG and GA were located at 3422.5, 3358, and 3406 cm^−1^, respectively. Obviously, in the spectra of Zein-EGCG and Zein-GA-EGCG complex nanoparticles, these absorption peaks moved to 3421, 3412, 3432, 3416, 3446, 3420 and 3424 cm^−1^, respectively. These results indicated that a hydrogen bonding interaction had occurred in the complex samples [30]. Dai et al. also observed similar results in the zein-lecithin complex loaded with curcumin [31].

The absorption peaks of zein at 1654 cm^−1^ and 1542 cm^−1^ represented the C=O stretching vibration (amide I band) and N-H bending vibration (amide II band), respectively [32,33]. In Zein-EGCG and Zein-GA-EGCG complex nanoparticles, these absorption peaks shifted, indicating that electrostatic interaction existed among zein, GA and EGCG. Furthermore, zein has highly hydrophobic molecules, indicating that hydrophobic interactions may be participating in the complex nanoparticle formation [34]. Notably, when zein and EGCG formed the nanoparticles, the characteristic peaks of EGCG disappeared, indicating that EGCG was embedded in the complex nanoparticles. Based on the above results, zein, GA, and EGCG formed stable complexes mainly based on hydrogen bonding, electrostatic and hydrophobic interactions. Interestingly, when EGCG was incorporated into the nanoparticles, the featured absorption bands observed in the spectrum of pure EGCG disappeared, which indicated that EGCG was encapsulated in the complex nanoparticles.

### 3.4. Analysis of XRD

**Figure 5:**
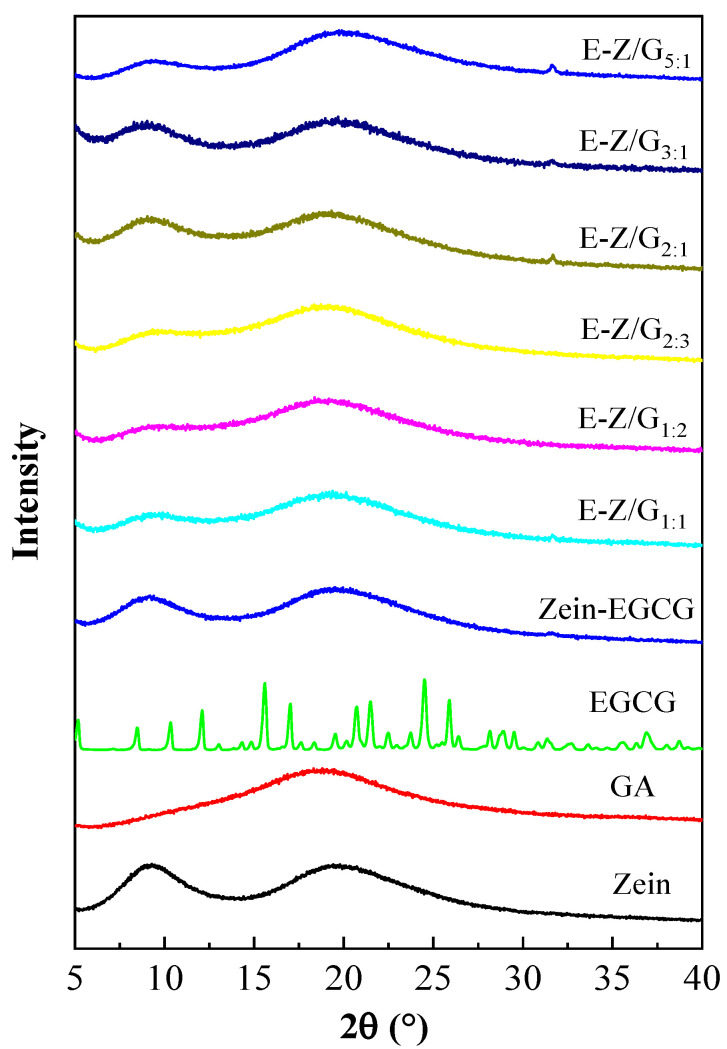
The XRD analysis of different samples.

The XRD results of zein, EGCG, GA and Zein-GA-EGCG samples with different mass ratios are shown in Figure 5. Two wide peaks around 10° and 20° were observed in the sample of zein, and one wide peak near 18° was observed in GA, indicating that zein and GA were in amorphous form. Significant characteristic spikes were observed at 15, 20 and 25° for the sample of EGCG, indicating that EGCG had the characteristics of crystal structure, which was in line with the study of Liang et al. [24]. When EGCG was encapsulated in Zein-GA complex, no characteristic peak of EGCG was observed in Zein-GA-EGCG, suggesting that EGCG was successfully encapsulated. Notably, the intensity of diffraction peak at 2θ = 10° of Zein-GA-EGCG complexes decreased with the increase in GA content, while the intensity of peaks near 2θ = 20° increased. These results proved that Zein-GA-EGCG complex nanoparticles were formed. Souza et al. observed a similar phenomenon in the ovalbumin-pectin complex [35].

### 3.5. Analysis of DSC

**Figure 6:**
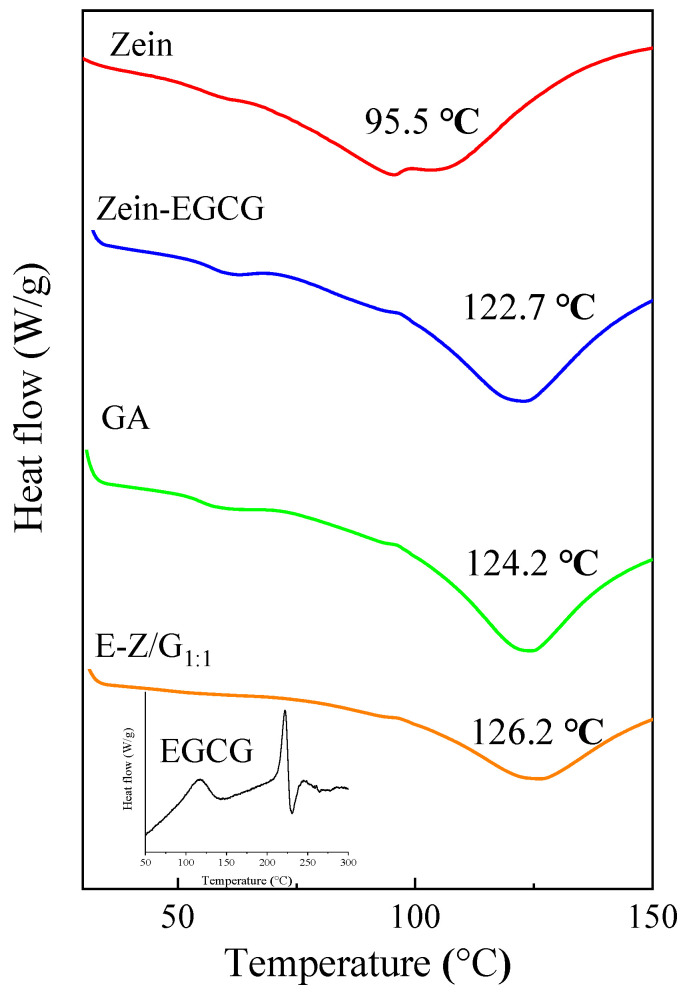
The DSC analysis of different samples.

The thermal stability of zein, GA, Zein-EGCG and E-Z/G_1:1_ complex nanoparticles was studied by a differential scanning calorimetry (DSC) (Figure 6). EGCG showed a sharp endothermic peak at 220 °C, which corresponded to the EGCG melting point [36]. Zein had an endothermic peak at 95.5 °C, while GA had a higher endothermic peak at 124.2 °C, resulting from the stronger affinity of polysaccharides to water [37]. Compared with zein alone, the endothermic peaks of Zein-EGCG and E-Z/G_1:1_ moved to 122.7 °C and 126.2 °C, respectively. The presence of EGCG and GA enhanced the interactions between different components in the nanocomposite, such as the hydrophobic and electrostatic interaction, resulting in an increase in the endothermic peak temperature [38]. Dai et al. observed a similar phenomenon in the zein-lecithin-curcumin system and pointed out that the addition of lecithin improved the thermal stability of the protein [30].

### 3.6. Free Radical Scavenging Ability Analysis of DPPH and ABTS

**Figure 7:**
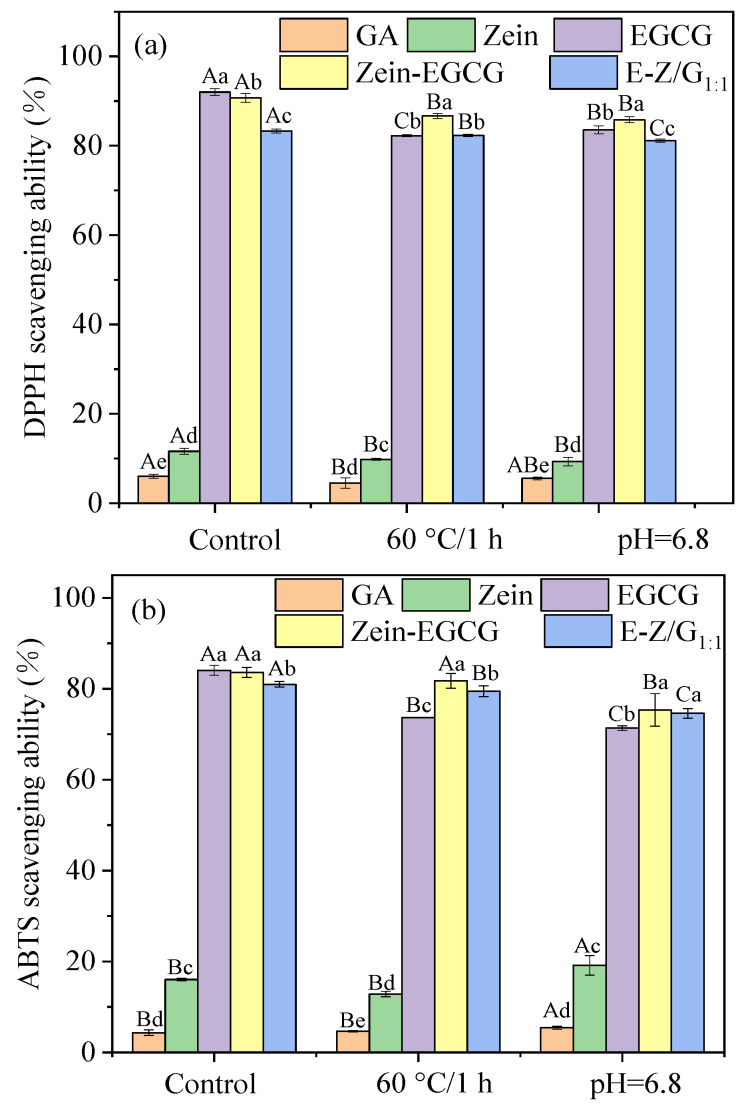
DPPH (**a**) and ABTS (**b**) radical scavenging capacity of different samples. The concentration of Zein, GA, and Zein-GA was 0.2% and the content of EGCG was 100 mg/L. Different lowercase letters represents the significant difference within groups and different uppercase letters represent a significant difference across groups.

The antioxidant activity of EGCG gives it its health values [39,40,41]. Both DPPH and ABTS methods were used because the antioxidant activity of active compounds could not be accurately determined by a single method. As shown in Figure 7, the antioxidant capacity of GA was negligible. Zein showed weak antioxidant capacity because some amino acids in natural proteins were reducible. With the change of pH and temperature, the antioxidant activities of GA and zein did not fluctuate greatly, while the scavenging ability of free EGCG on DPPH radical decreased. This may be due to the instability of EGCG at high temperature and in neutral pH [42,43]. Notably, the antioxidant capacity of E-Z/G_1:1_ complex nanoparticles also decreased, but the reduction was less than that of pure EGCG. These results indicated that EGCG had stronger non-covalent binding energy with zein and GA, which can scavenge free radicals and form more stable complexes. In addition, the trends of ABTS results were consistent with those of DPPH. The EGCG contained two adjacent hydroxyl groups on the aromatic ring, and the EGCG molecule was not destroyed during the formation of the complexes, and the hydroxyl group on the second ring may still remain free [44]. Thus, the antioxidant activity of EGCG was still strong. Therefore, Zein-GA-EGCG complex nanoparticles can be used as an effective antioxidant in food systems.

### 3.7. Release of EGCG during Simulated Digestion

**Figure 8:**
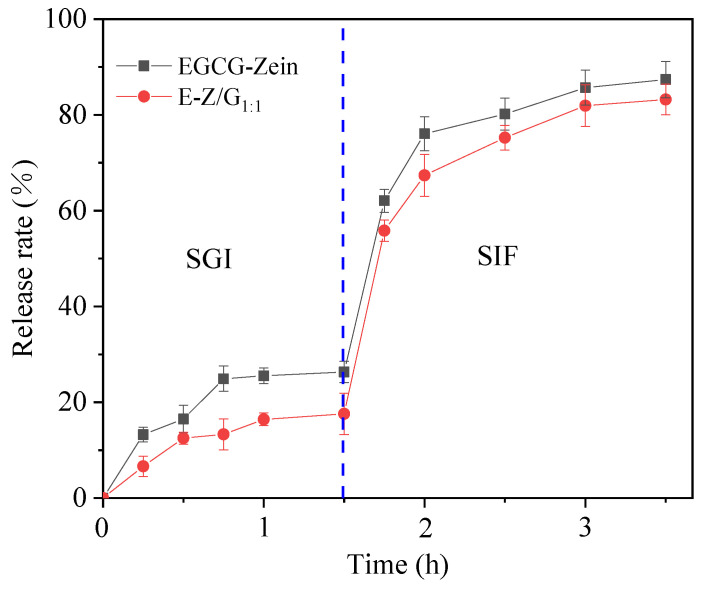
The cumulative release rate of EGCG in simulated gastric fluid (SGF) and simulated intestinal fluid (SIF).

The release rate of EGCG in Zein-EGCG and E-Z/G complex nanoparticles was very low during the simulated gastric digestion (Figure 8). The EGCG release rate of E-Z/G_1:1_ complex nanoparticles (16.42%) was significantly lower than that of Zein-EGCG complex nanoparticles (25.52%) after gastric digestion for 1.5 h. This might be caused by the presence of GA, which prevented EGCG from release. In addition, the interaction between zein, GA and EGCG made the complex nanoparticles become more compact, reducing the damage of pepsin to protein, and inhibiting the release of EGCG from the complex nanoparticles [45,46,47]. When the sample was transferred from simulated gastric fluid to simulated intestinal fluid, the release rate of EGCG increased rapidly in the first hour and then became stabilized. At the end of intestinal digestion, the values of release rate of EGCG from Zein-EGCG and E-Z/G_1:1_ complex nanoparticles reached 87.38 and 84.23%, respectively. This may be because the interaction between GA and zein was weakened with the change of environmental pH, allowing trypsin to damage the structure of zein and resulting in the massive release of EGCG [48,49,50].

**Figure 9:**
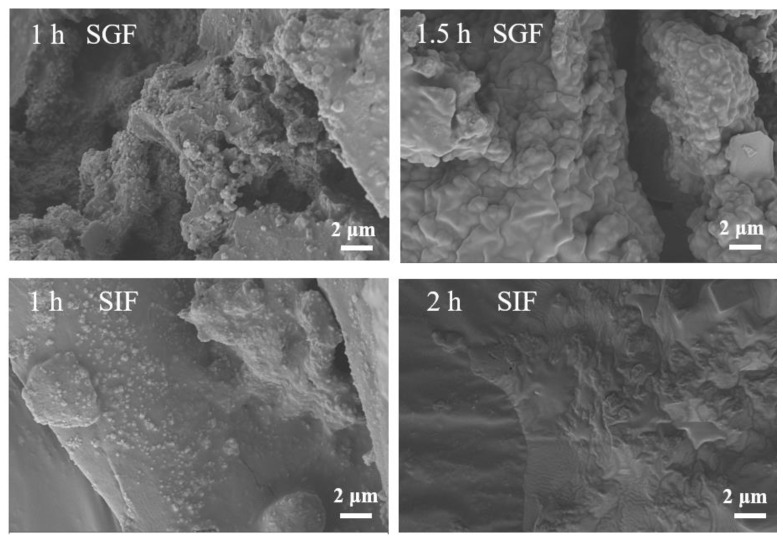
SEM images of E-Z/G_1:1_ complex nanoparticles in simulated gastric fluid (SGF) and simulated intestinal fluid (SIF).

Figure 9 shows the micromorphology of E-Z/G_1:1_ samples at different digestion times. The morphology of these nanoparticles changed significantly after simulated gastrointestinal digestion (Figure 9). The complex nanoparticles still maintained their particle shape after being digested in SGF for 1 h. Then, the complex nanoparticles appeared to show adhesion and swelling at the end of gastric digestion (1.5 h). The particle shape of the nanocomposite gradually disappeared during simulated intestinal digestion due to the shearing action of trypsin. Structural damage of the proteins led to the release of EGCG into the intestinal fluid. These results suggested that E-Z/G_1:1_ complex nanoparticles can effectively reduce the degradation of EGCG and achieve targeted release under simulated gastrointestinal conditions. Huang et al. also pointed out that a lysozyme-carrageenan complex could greatly protect curcumin from digestion and degradation, and provide a good barrier for the release of curcumin [51,52].

## 4. Conclusions

In this study, a Zein-GA composite system was constructed to encapsulate and protect EGCG. The particle size of the Zein-GA-EGCG complex nanoparticles ranged from 128.03–221.23 nm, and the encapsulation efficiency of EGCG reached a maximum of 75.23% in E-Z/G_1:1_ sample. FTIR and XRD were used to investigate the binding mechanism of the Zein-GA-EGCG complex nanoparticles. The results showed that EGCG can exist in complex nanoparticles in an amorphous state, and that hydrogen bonding, hydrophobic and electrostatic interactions existed in the complex nanoparticles. DSC, ABTS and DPPH experiments showed that the complex samples had high thermal stability and good antioxidant activity. The sustained release behavior of the EGCG in the Zein-GA-EGCG complex nanoparticles was investigated in vitro. The EGCG release rate of the Zein-GA-EGCG nanoparticles (16.42%) was lower than that of the Zein-EGCG (25.52%) after gastric digestion, and a large amount of EGCG was released during intestinal digestion, suggesting that Zein-GA-EGCG nanoparticles could achieve sustained release of EGCG during in vitro digestion.

## Data Availability

The data presented in this study are available on request from the corresponding author.
